# Cancer-associated fibroblasts support vascular growth through mechanical force

**DOI:** 10.1038/s41598-017-13006-x

**Published:** 2017-10-03

**Authors:** Mary Kathryn Sewell-Loftin, Samantha Van Hove Bayer, Elizabeth Crist, Taylor Hughes, Sofia M. Joison, Gregory D. Longmore, Steven C. George

**Affiliations:** 1 Departments of Biomedical Engineering, Washington University in St. Louis, St. Louis, MO 63130 USA; 2 Departments of Cell Biology and Physiology, Washington University in St. Louis, St. Louis, MO 63130 USA; 3 Departments of Computer Science and Engineering, Washington University in St. Louis, St. Louis, MO 63130 USA; 4 Department of Medicine, Oncology Division, Washington University in St. Louis, St. Louis, MO 63110 USA; 5 ICCE Institute at Washington University, Washington University in St. Louis, St. Louis, MO 63110 USA; 6 Department of Biomedical Engineering, University of California, Davis, CA 95616 USA

## Abstract

The role of cancer-associated fibroblasts (CAFs) as regulators of tumor progression, specifically vascular growth, has only recently been described. CAFs are thought to be more mechanically active but how this trait may alter the tumor microenvironment is poorly understood. We hypothesized that enhanced mechanical activity of CAFs, as regulated by the Rho/ROCK pathway, contributes to increased blood vessel growth. Using a 3D *in vitro* tissue model of vasculogenesis, we observed increased vascularization in the presence of breast cancer CAFs compared to normal breast fibroblasts. Further studies indicated this phenomenon was not simply a result of enhanced soluble signaling factors, including vascular endothelial growth factor (VEGF), and that CAFs generated significantly larger deformations in 3D gels compared to normal fibroblasts. Inhibition of the mechanotransductive pathways abrogated the ability of CAFs to deform the matrix and suppressed vascularization. Finally, utilizing magnetic microbeads to mechanically stimulate mechanically-inhibited CAFs showed partial rescue of vascularization. Our studies demonstrate enhanced mechanical activity of CAFs may play a crucial and previously unappreciated role in the formation of tumor-associated vasculature which could possibly offer potential novel targets in future anti-cancer therapies.

## Introduction

The formation of new blood vessels in the tumor microenvironment is essential for tumor progression and generally occurs when the metabolic demands of the tumor exceed the existing supply. Although blood vessel growth is an attractive target for anti-cancer treatments^1,2^, anti-angiogenic therapies that target vascular endothelial growth factor (VEGF) and its receptors (VEGFRs) have demonstrated limited efficacy in numerous tumor types^3,4^. This may be due, in part, to our limited understanding of other factors, such as the mechanical properties of the tumor microenvironment, in controlling cancer associated vascular growth. Mechanical force can be both normal and parallel to a substrate (or cell) and are generally referred to as tensile/compressive and shear, respectively. Previous work has demonstrated that the mechanical force of shear flow can drive vascular growth^5–7^, but there is limited work describing how tensile forces may regulate vascular growth^8,9^.

Recently, cancer-associated fibroblasts (CAFs) have been described as potent regulators of tumor progression, including angiogenesis and metastasis^10–16^. CAFs are a heterogeneous population of stromal cells present in the tumor microenvironment that can be derived from several tissue sources^17,18^. There are no significant somatic differences between CAFs and their normal counterparts, indicating that oncogenic mutations do not contribute to the CAF phenotype^19^. CAFs have a myofibroblast-like phenotype, characterized by enhanced secretory functions and mechanical activity compared to resident fibroblasts. For example, CAFs secrete elevated levels of stromal derived factor 1 (SDF1) and VEGF^20–22^, as well as demonstrate increased tensile forces potentially mediated through the YAP-pathway^23,24^. While CAF signaling pathways, including VEGF and hypoxia inducible factor 1-alpha (HIF-1α) have been extensively studied, the role of mechanics in tumor progression specifically CAF-generated mechanical forces is poorly understood^20,25,26^. We hypothesized that CAF-mediated mechanical forces may contribute to enhanced vascular growth in the tumor microenvironment in a way that is complementary to, but distinct from, VEGF signaling.

Using a 3D *in vitro* model of blood vessel formation, we demonstrate that CAFs, derived from human breast cancer^27^, support the self-assembly of vascular networks when co-cultured with endothelial cells in fibrin hydrogels. Similar vascular growth is not seen in samples containing normal breast fibroblasts (NBFs) and endothelial cells. Further studies demonstrate that CAF-supported vessel growth is not solely dependent on soluble factors, including VEGF, but that inhibition of mechanotransductive pathways including ROCK, YAP, and Snail1 (SN1) in the CAF attenuates vascular growth. Finally, inducing mechanical perturbations with magnetic beads in the absence of CAF-generated extracellular matrix (ECM) deformations and upregulating the mechanical activity of NBFs both stimulate blood vessel growth. Additionally, we examined the mechanical activity of normal human lung fibroblasts (NHLFs), which have been shown by our lab to be effective stromal cells for vascularization studies^28–32^. Studies showed similar results to CAF vascularization potential and biomechanical behaviors, indicating that mechanobiology may regulate vascular growth in noncancerous environments as well. Taken together, our data demonstrate that CAF-mediated biomechanical activity can regulate vascular growth and might contribute to blood vessel formation in the tumor microenvironment.

## Results

### CAFs support vascularization in 3D hydrogels

**Figure 1:**
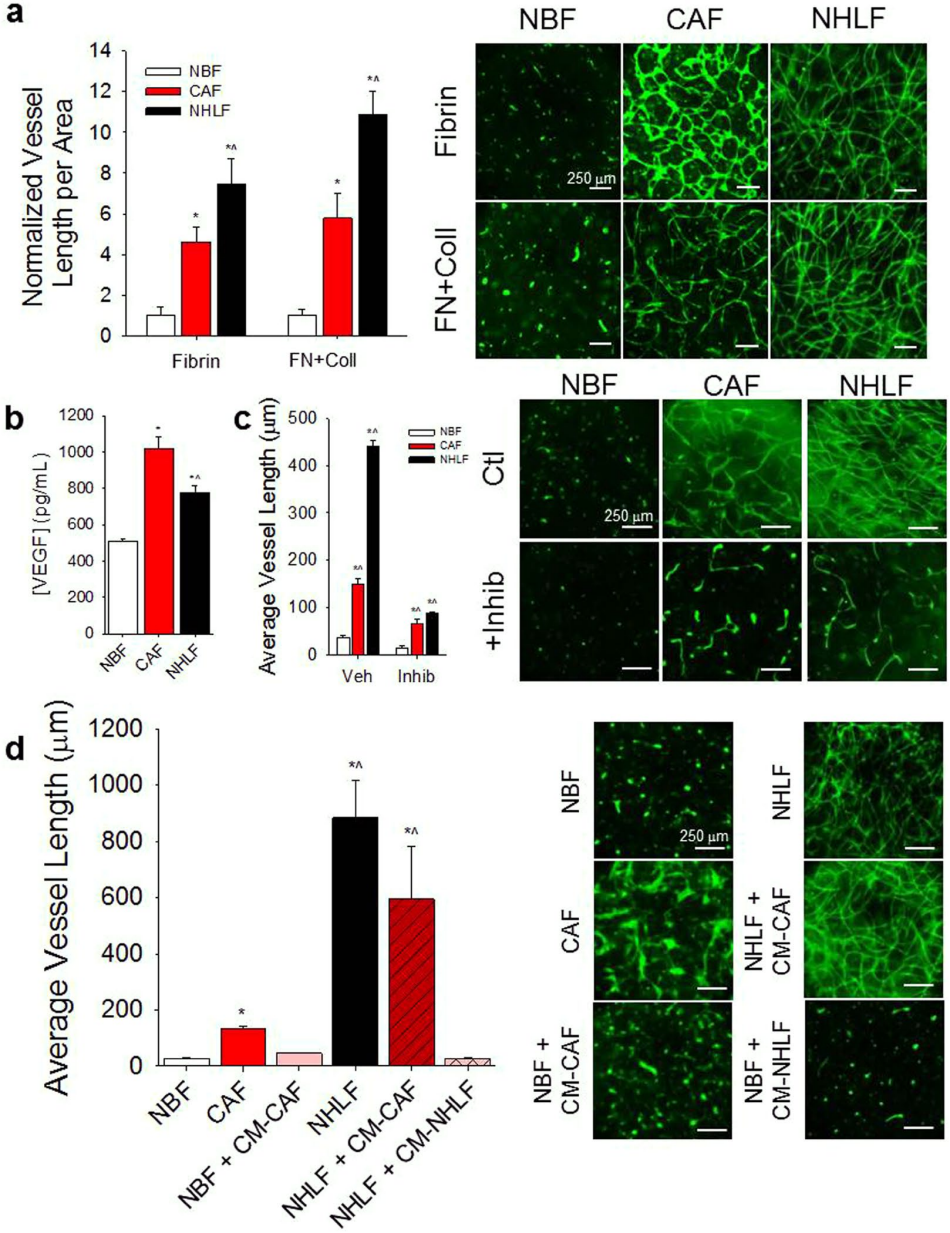
CAFs support vascularization in 3D microtissues. (**a**) When co-cultured with ECs in Fibrin or combination Fibrin-Collagen (FN + Coll) gels, CAFs support significantly more vascular growth compared to NBFs. NHLF also demonstrate significantly higher vascularization potential compared to NBFs. Data are presented as total vessel length per unit area, normalized to NBF in Fibrin: 0.0014 ± 0.0002 μm^−1^; or NBF in FN + Coll: 0.0018 ± 0.0006 μm^−1^. *p < 0.01 vs. NBF; ^p < 0.01 vs. CAF for same gel type. (Right) Immunofluorescent images of CD31 staining of 3D vessel systems show interconnected vascular networks in CAF & NHLF samples, but not in NBF samples. (**b**) CAFs in co-culture with ECs (CAFs/ECs) demonstrate higher steady state levels of soluble VEGF than NBFs/ECs. NHLF/EC co-cultures exhibit significantly lower levels of VEGF compared to CAF/EC samples. *p < 0.01 vs. NBF; ^p < 0.01 vs. CAF. (**c**) Inhibition of VEGFRs suppresses CAF- and NHLF-supported vascular growth compared to vehicle treated controls but shows significantly larger average vessel growth compared to NBF vehicle controls. Data are presented as average vessel length in μm. *p < 0.01 vs. NBF vehicle; ^p < 0.01 vs. NBF + inhibitor (Right) Immunofluorescent images of CD31 staining show vascular fragments of >100 μm in length present in CAF samples with inhibited VEGFR. (**d**) Conditioned media from CAF/EC cultures (CM-CAF) minimally rescues vascular formation in fibrin gels. Conditioned medial from NHLF/EC (CM-NHLF) cultures showed similar results when added to NBF samples. Data are presented as average vessel length in μm. (Right) Immunofluorescent staining of gels for CD31 demonstrate NBF + CM-CAF or + CM-NHLF groups exhibit only short fragments of blood vessels, <50 μm in length. *p < 0.01 vs. NBF; ^p < 0.01 vs. CAF. Scale bars = 250 μm.

When co-cultured with endothelial cells (ECs), CAFs support the formation of interconnected vascular networks in 3D fibrin and fibrin-collagen gels (Fig. 1a). In contrast, NBFs do not support vessel network formation in the same gel compositions. The addition of collagen to fibrin gels does not significantly alter vessel growth supported by CAFs, NBFs, or NHLFs. Collagen-only gels were not utilized, as compaction occurs too quickly in these samples to permit observable vascularization.

### Soluble factors partially account for enhanced vessel growth

Media from CAF/EC and NHLF/EC cultures exhibit approximately twice the steady state level of VEGF compared to NBF/EC (Fig. 1b). A small molecule inhibitor of VEGFRs and fibroblast growth factor receptors (SU-5402, Abcam) partially suppressed CAF- and NHLF-mediated vessel formation (Fig. 1c), however the average vessel length in inhibited CAF samples is significantly higher than vessel lengths observed in NBF vehicle treated samples. Average vessel length in inhibited NHLF samples was also significantly higher than the NBF vehicle sample group. When conditioned media from a co-culture of CAFs/ECs (CAF-CM) that produced vessels was supplied to a co-culture of NBFs/ECs and analyzed for vessel growth after 7 days, vessel growth was stimulated but significantly less than CAF control samples (Fig. 1d). Furthermore, the fluorescent images show only short vessel fragments <200 μm in length in the CAF-CM treated samples. NHLF/EC conditioned media (CM-NHLF) was also unable to produce increases in vascularization of NBF/EC samples (Fig. 1d). These data demonstrate that soluble mediators, including VEGF, are not solely responsible for increases in vascularization potential demonstrated by CAFs in 3D gels. We next chose to investigate the mechanosensitive properties of CAFs as a potential explanation for this behaviour.

### CAFs vascularization potential is enhanced by fibrin density

**Figure 2:**
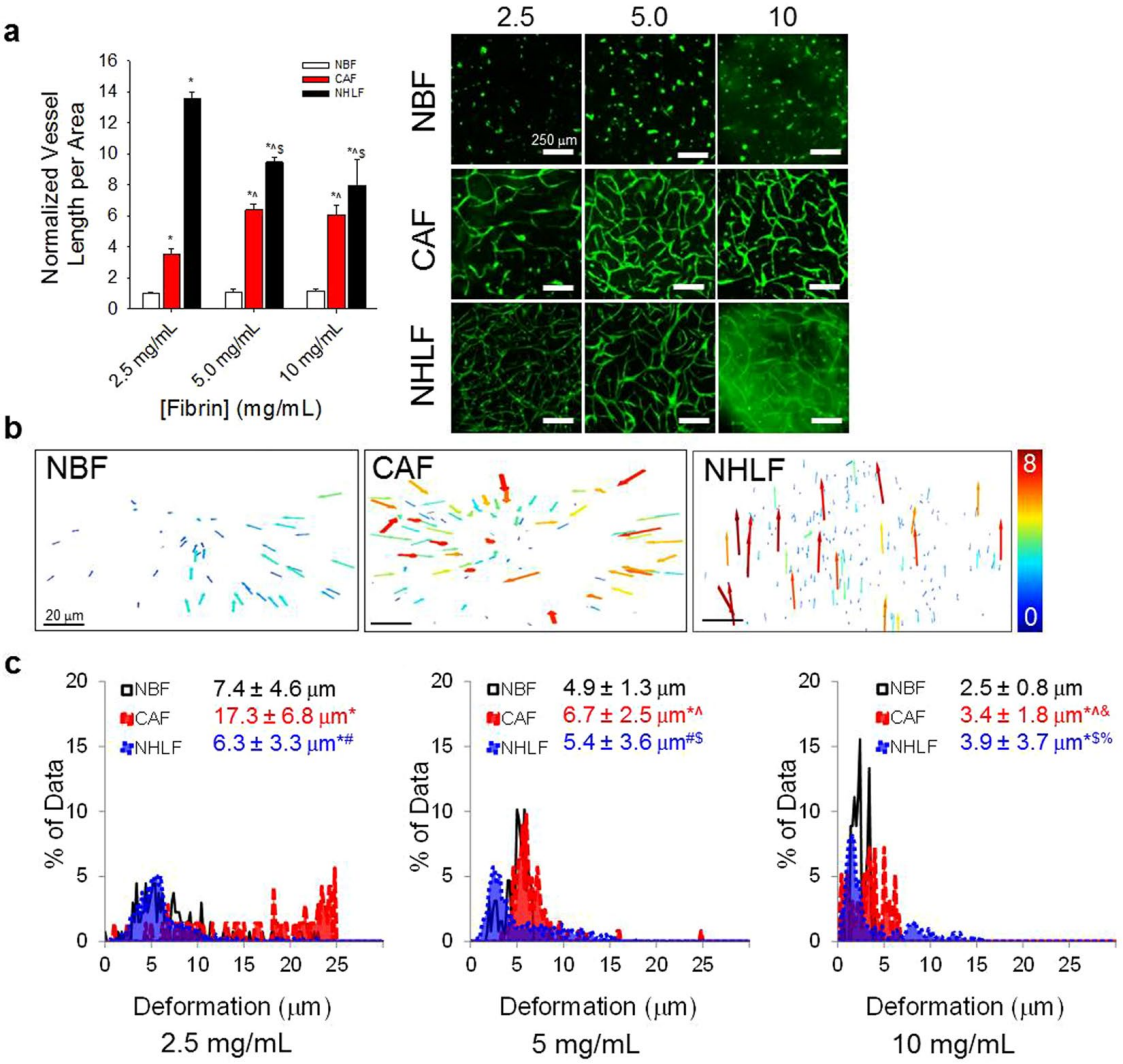
CAFs demonstrate mechanosensitive vascularization potential. (**a**) CAFs support enhanced levels of vascular growth over increasing fibrin gel compositions, with a significant increase seen from 2.5 mg/mL to 5.0 mg/mL samples. NHLF samples demonstrate the opposite effect, with significantly decreased vascularization potential as fibrin concentration increases over the same range. Data are presented as total vessel length per area, normalized to NBF samples in 2.5 mg/mL fibrin gels: 0.0017 ± 0.0001 μm^−1^. (Right) Immunofluorescent images of CD31 staining show vascular networks formed in the presence of CAFs and NHLFs, but not NBFs. *p < 0.01 vs. NBF at same fibrin concentration; ^p < 0.01 vs. CAF at 2.5 mg/mL fibrin; ^$^p < 0.01 vs. NHLF at 2.5 mg/mL. Scale bars = 250 μm. (**b**) CAFs generate larger deformations in 3D gels compared to NBFs, as seen in 3D vector plots of bead displacements. Each arrow represents a tracked movement of a fiducial marker; color corresponds to magnitude as indicated by the scale bar (0–8 μm). Inset scale bars = 20 μm. (**c**) Data from vector maps is pooled from a minimum of 2 technical replicates, binned and plotted as histograms to show population behavior, where the y-axis represents the percent of data collected at a particular deformation value. Inset numbers are average deformation magnitudes ± standard deviation for each cell line. Each plot represents n > 40 bead movements tracked. CAFs demonstrate significantly larger deformations at all gel compositions compared to NBFs. CAFs have significantly larger averaged deformations compared to NHLFs at 2.5 and 5.0 mg/mL fibrin only. NHLFs exhibit larger deformations compared to NBFs in 5.0 mg/mL and 10 mg/mL fibrin gels,. *p < 0.01 vs. NBF at same fibrin concentration for average values; # vs. CAF at same fibrin concentration; ^p < 0.01 vs. CAF at 2.5 mg/mL fibrin; ^&^p < 0.01 vs. CAF at 5.0 mg/mL; ^$^p < 0.01 vs. NHLF at 2.5 mg/mL fibrin; ^%^p < 0.01 vs. NHLF at 5.0 mg/mL fibrin. More statistical data can be found in Table S1.

The ability for CAFs to promote blood vessel growth was significantly enhanced by increasing fibrin concentration from 2.5 mg/L to 5.0 mg/mL (Fig. 2a). Above this, a plateau of vessel growth was reached. There were no changes in vessel network formation promoted by NBFs at any of the fibrin concentrations tested. NHLF samples actually demonstrated a significant decrease in vascular growth as concentration of fibrin increased.

### CAFs generate larger ECM deformations in 3D

Using our custom bead-tracking algorithm, we measured displacements in 3D fibrin gels in the presence of NHLFs, NBFs or CAFs. At all concentrations of fibrin gels tested, CAFs produce significantly higher mean deformations in the ECM compared to NBFs (Fig. 2b,c). Previous work from our lab has measured the compressive modulus of these fibrin gels at approximately 1 kPa (2.5 mg/mL), 4 kPa (5.0 mg/mL), and 9 kPa (10 mg/mL)^33^. Increasing the fibrin concentration, or effective stiffness of the gels, signficantly decreases the magnitude of displacements for all cell types. Average deformation values and more complete statistical data can be found in Supplemental Table S1.

Based on our experiments, we observed some changes in vascularization potential and mechanical behavior of CAFs in response to changes in mechanical properties of the ECM. This aligns with previous results in the literature, showing CAFs have a more myofibroblast-like phenotype including enhanced contractility in 2D^23,24^. Additionally, our deformations measurements highlight the heterogeneity of biomechanical properties of CAF behaviors. These results indicate that the biomechanical activity of CAFs may play a role in the development of blood vessel networks and promoted further investigations.

### Interfering with CAF mechanotransduction alters vasculogenic potential

**Figure 3:**
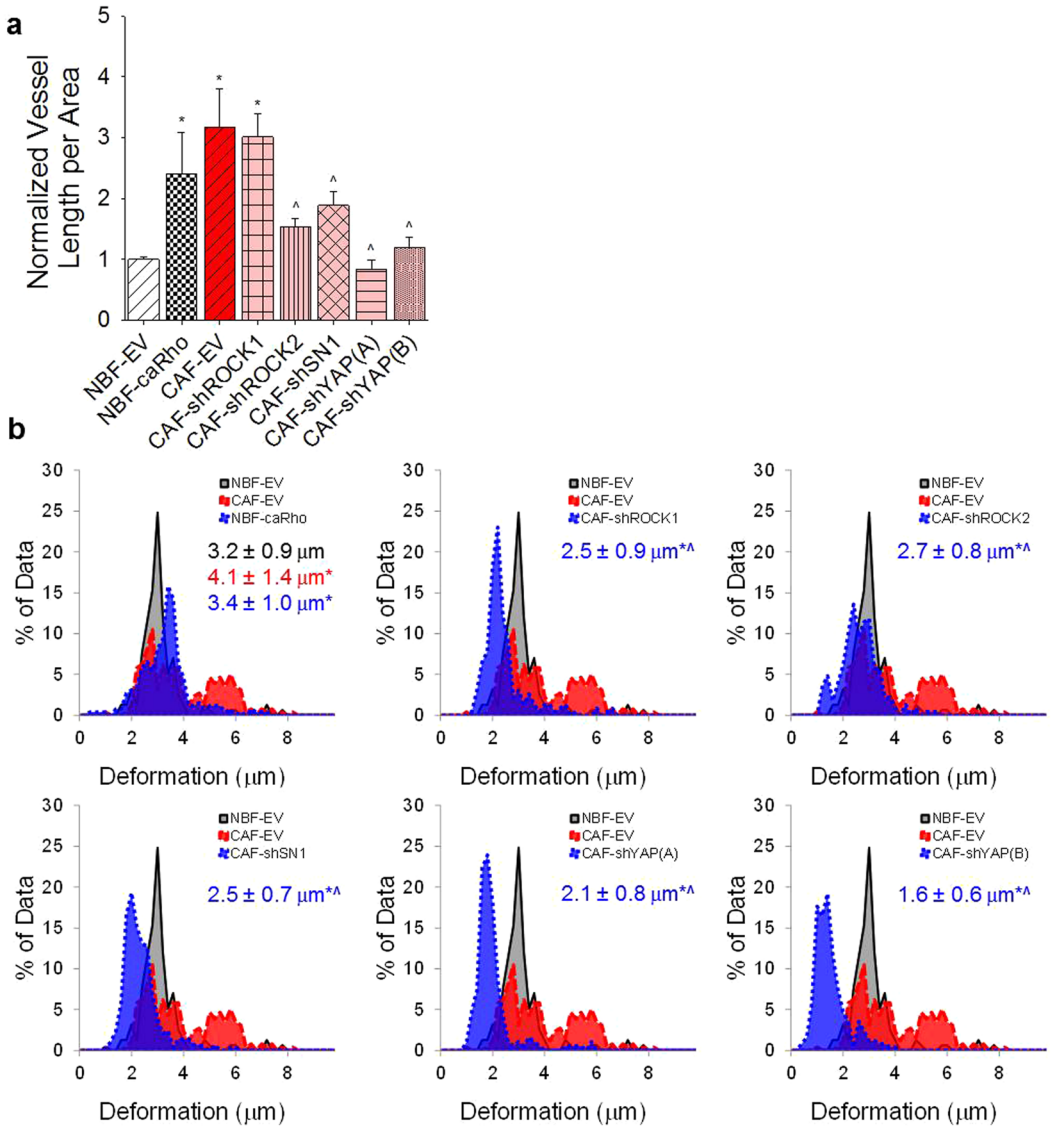
Mechanotransductive pathways regulate CAF supported vascular growth. (**a**) Inserting caRho into NBFs promotes enhanced blood vessel growth compared to NBF empty vector (EV) controls. Furthermore, inhibiting ROCK2, SN1, or YAP in CAFs suppresses formation of blood vessels in 3D cultures compared to CAF-EVs. Inhibition of ROCK1 in CAFs produced no significant changes in vascularization measured. Data are presented as total vessel length per area normalized to NBF-EV: 0.0032 ± 0.0001 μm^−1^. *p < 0.05 versus NBF-EV; ^p < 0.05 versus CAF-EV. (**b**) Histograms showing distribution of deformations induced by genetically-modified fibroblasts in 3D fibrin gels. Inset numbers are average deformation magnitudes ± standard deviation for each cell line. NBF-EV and CAF-EV data are shown on all plots to facilitate ease in comparing inhibited lines to controls. The addition of caRho shifts the peak of observed deformations to the right, indicating higher levels of mechanical activity in these cells compared to NBF-EVs. Alternatively, inhibiting ROCK, SN1, and YAP shifts peak deformations to the left, demonstrating that the modified CAFs generate smaller deformations in 3D compared to CAF-EVs. *p < 0.05 vs. NBF-EV; ^p < 0.05 vs. CAF-EV.

Previous work has shown that ROCK, YAP, and SN1 are all parts of the mechanotransductive pathways of CAFs, regulating how the cells interact with the matrix surrounding them. Utilizing shRNA to inhibit YAP and SN1 pathway inhibited the ability of CAFs to support blood vessel formation in 3D compared to CAF empty vector (EV) controls (Fig. 3a). Interestingly, inhibition of ROCK1 had no effect on this behavior while knockdown of ROCK2 significantly suppressed vascular formation supported by CAF-EVs. Furthermore, a constitutively active form of Rho (caRho) inserted into NBFs demonstrated enhanced vascular network formation compared to NBF-EV controls, comparable to that of CAF-EV samples. Bead displacement studies show CAF-EVs are significantly more contractile than NBF-EVs and that inhibition of mechanotransductive pathways abrogates the 3D deformations generated by CAF-based lines (Fig. 3b). Samples containing inhibited ROCK1 and ROCK2 showed similar average deformations, but dramatically different vascular growth; this may be due in part to differences in localization of ROCK1 versus ROCK2 inside of CAFs during mechanotransductive events. Furthermore, ability to deform the matrix alone may not be sufficient to drive vascularization. As expected, NBF-caRho samples demonstrated significantly larger deformations than NBF-EV and were similar to those generated by CAF-EVs.

**Figure 4:**
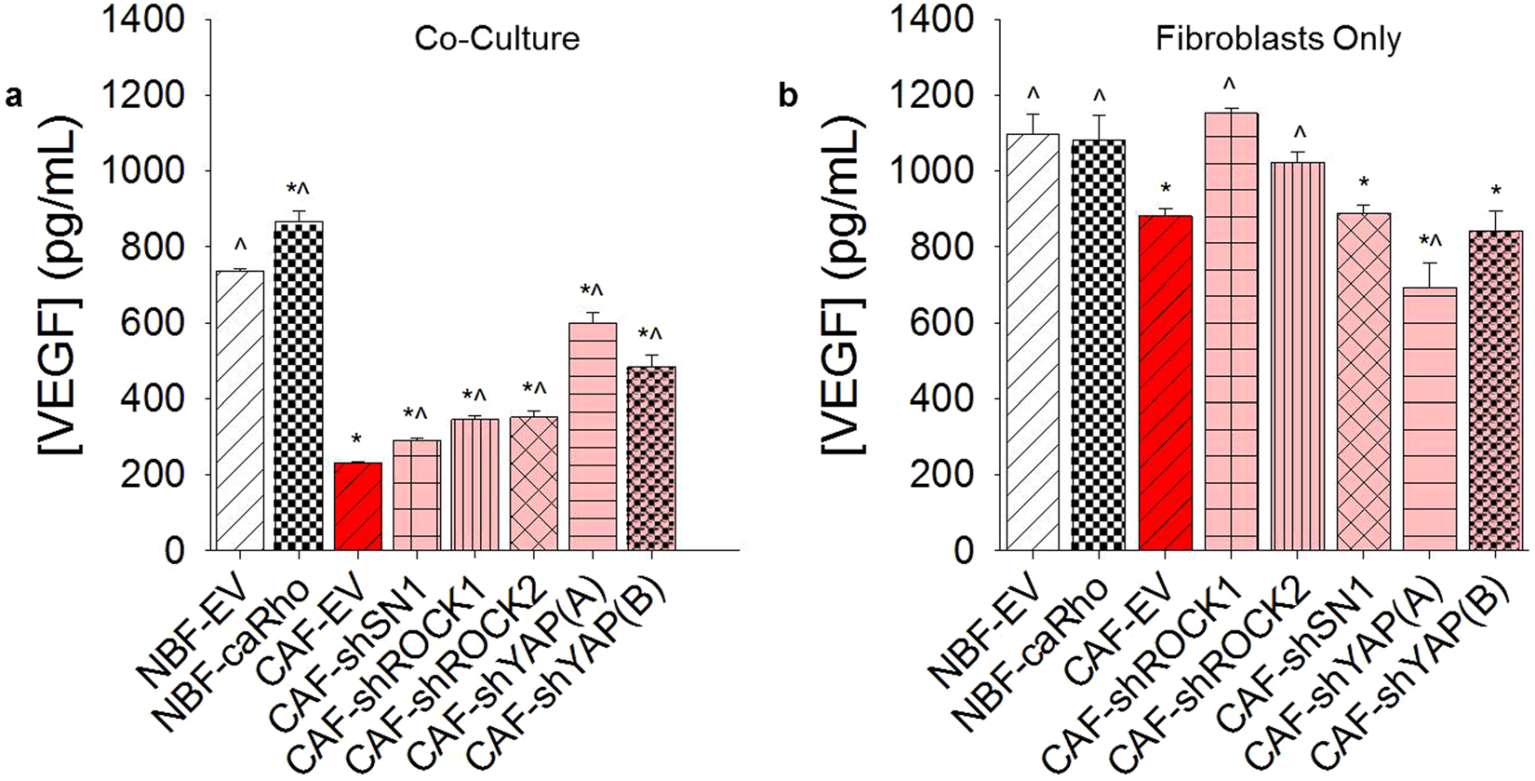
VEGF production and utilization is impacted by mechanotransduction inhibition. (**a**) VEGF produced during the vasculogenic ring assay demonstrates differences between EV controls and modified cells lines. *p < 0.05 vs. NBF-EV/EC; ^p < 0.05 vs. CAF-EV/EC. Increased levels of VEGF do not necessarily correspond to increased vascular growth, as demonstrated by NBF-EV/EC samples having significantly higher steady state levels of VEGF than CAF-EV/EC samples. (**b**) To determine how EC presence affected VEGF measurements in the ring assay, we measured VEGF production in fibroblast only cultures in the same concentration 3D fibrin gels. These results suggest that modified CAFs may produce more VEGF than CAF-EVs, but that the ECs in these cultures are less efficient at utilizing the molecule for the creation of vessels. Further, there is no difference in NBF-EV and NBF-caRho fibroblast only samples measurements of VEGF, while NBF-caRho/EC samples show more VEGF than NBF-EV/EC samples. This indicates that VEGF consumption alone cannot explain enhanced vessel growth in NBF-caRho/EC samples. *p < 0.05 versus NBF-EV; ^p < 0.05 vs. CAF-EV.

We measured the VEGF concentration in 3D fibroblast only cultures to determine how the presence of ECs might affect overall steady state VEGF levels. Based on this comparison, we hypothesize that mechanical activity of CAF-EVs may promote ECs present in co-cultures to consume or utilize more VEGF, resulting in lower measured steady state levels when compared to modified CAF/EC cultures (Fig. 4a). Since our studies do not specifically elucidate differences in VEGF production or consumption, this hypothesis attempts to address why modified NBF samples have higher levels of VEGF compared to modified CAF lines. As modified CAF samples seem to produce either less or more VEGF (depending on targeted sequence) compared to CAF-EV controls, we conclude that steady state VEGF levels alone are not sufficient to explain CAF driven vascular growth (Fig. 4b). This is demonstrated by the fact that CAF-shROCK1/EC and CAF-shROCK2/EC samples produce similar steady levels of VEGF but different levels of vascular growth. Furthermore, there is no significant difference in VEGF secreted by NBF-EV and NBF-caRho cells in 3D, while co-culturing with ECs produces reduced VEGF readings in NBF-EV samples. Taken together, we believe this further supports our hypothesis that mechanical activity of CAFs modulates vascular growth in a manner not solely dependent on the VEGF pathway.

These results demonstrate that biomechanical activity induced in the ECM by CAF mechanotransduction regulated by ROCK2, SN1, and YAP are important in the regulation of CAF promoted blood vessel growth. Furthermore, enhancing mechanical perturbations to the microenvironment by constitutively activating Rho in NBFs generated greater vascularization in these samples, representing nearly a 2-fold increase in vascularization potential. Moving forward, we chose to utilize a magnet-based system to physically induce mechanical deformations in the microenvironment regardless of the presence of stromal-secreted factors.

### Magnetic stimulation partially recapitulates vessel growth

**Figure 5:**
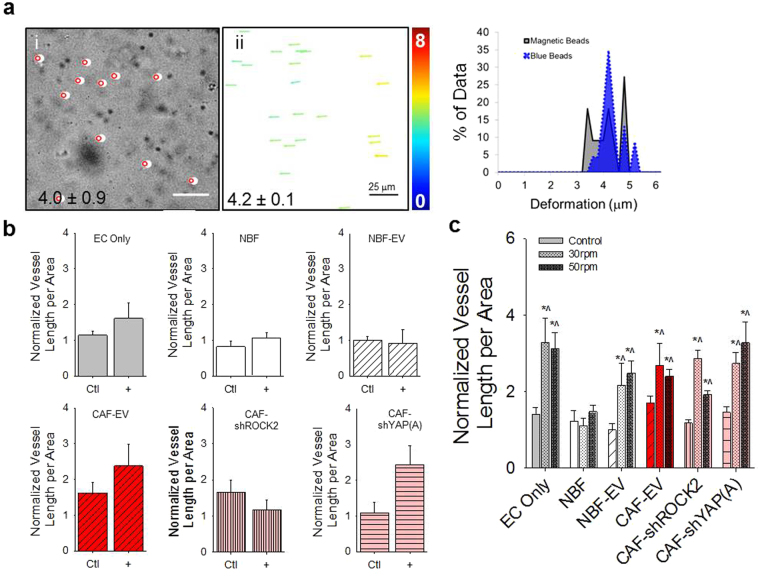
Magnetic induction of ECM deformations partially rescues vascularization. (**a**) Thrombin-coated magnetic beads move in response to magnetic stimulation; magnet was located on left of images shown (i). In bright field images, magnetic bead movement can be easily tracked. White circles indicate an initial position; red circles indicate final position. Inset number is average movement ± standard error of the mean (ii). For same region shown in (i), blue 1 μm fluorescent beads were tracked using the MATLAB bead tracking algorithm described in this paper. Color bar represents magnitude of displacement (0–8 μm). Inset number is average movement ± standard error of the mean. There is statistically no difference in the measured displacements (p = 0.775). Scale bars = 25 μm. (**b**) In a static study, a magnet was placed 2.5 cm to the left of fibrin gels containing thrombin-coated mangetic beads cultured with ECs only or with ECs and fibroblasts. No significant enhancement of blood vessel growth was observed in any of the samples, demonstrating that static strains induced the ECM are not sufficient to rescue vascularization. Vascular growth numbers are presented as total vessel length pre area normalized to NBF-EV control: 0.0038 ± 0.0013 μm^−1^. (**c**) In a dynamic stimulation study, thrombin-coated magnetic beads in fibrin gels containing ECs only or ECs and fibroblasts were moved by placing a N52 magnet on an orbital shaker located 3 cm below the gels. Magnetic bead movement stimulated enhanced blood vessel growth in all samples, except NBFs. This demonstrates mechanical activity in the ECM can moderately promote or rescue blood vessel growth in some samples that do not support vascularization in control studies. Vascular growth numbers are shown as total vessel length per area normalized to NBF-EV control: 0.0033 ± 0.0006 μm^−1^. *p < 0.05 versus NBF-EV control; ^p < 0.05 versus control group for each specific line.

Magnetic microbeads coated with thrombin were embedded in fibrin gels to provide a direct means of mechanically perturbing cells in the absence of factors secreted by CAFs. Thrombin-coated beads generate ECM deformations in the presence of a magnetic field (Fig. 5a–i) and these are congruent with bead displacement as measured by our MATLAB algorithm for tracking cell-induced deformations (Fig. 5a-ii). Static magnetic stimulation, where the magnet is placed adjacent to the microtissue for the duration of the experiment, shows a slight trend to increase vascular formation in most samples that previously were unable to support vessel formation (Fig. 5b). The exception is the CAF-shROCK2 with ECs group, which demonstrated no change. In dynamic movement studies, the addition of magnetic beads and moving magnetic field at 30 or 50 rpm demonstrated an increase in blood vessel growth supported in samples containing either ECs only or ECs in combination with NBF-EVs, CAF-shROCK2, or CAF-shYAP(A) (Fig. 5c). Increasing rotation speed to 100 rpm showed no increase in supported vessel growth (Fig. S1).

These studies support our hypothesis that mechanical deformations or perturbations of the ECM generated by cells regulate the growth of blood vessels as promoted by CAFs in the tumor microenvironment. In the absence of any stromal cells, EC only groups demonstrated ~3-fold increases in blood vessel growth stimulated by dynamic magnetic bead movements. In mechanically inhibited CAF samples, we were able to rescue the vascularization potential with dynamic magnetic stimulation. Overall, we believe this study demonstrates the role of biomechanical activity of CAFs as a potent regulator of vascular growth in an *in vitro* tumor microenvironment model. This biomechanical regulation occurs in concert with soluble signaling cues, including VEGF, to promote vascularization associated with tumor progression.

## Discussion

Our study demonstrates a role for CAF mechanics in the development of vasculature using an *in vitro* 3D microtissue model. Our results demonstrate that CAFs generate larger deformations within the ECM, compared to NBFs, which stimulate self-assembly of ECs into a vascular network. This process is mediated via mechanotransductive pathways in the CAF including ROCK2, YAP, and SN1. While VEGF signaling is important, the inhibition of VEGF receptors only partially abrogates the CAF-promoted vascular growth. Furthermore, CAF conditioned media only moderately rescues blood vessel growth in NBF samples, demonstrating that soluble factors alone are not sufficient to explain the enhanced blood vessel growth in the presence of CAFs. These conclusions are further supported by NHLF studies, showing that the mechanical activity of these cells correlates with blood vessel growth in fibrin gels and that VEGFR inhibition does not completely abrogate vessel growth; this work suggests that fibroblast mechanobiology may regulate vascularization in contexts other than cancer biology. On the other hand, our data also show that NHLFs generate smaller deformations in 2.5 mg/mL fibrin gels compared to NBFs, despite also showing approximately 10-fold higher level of vascularization potential. We believe this indicates that large mechanical deformations alone are insufficient to drive blood vessel growth supported by stromal cells and that it is an important interaction between biomechanics and soluble signaling factors that ultimately directs vasculogenic processes. Taken together, this enhanced understanding of CAF-mediated vascular network formation could potentially provide alternative therapeutic strategies that focus on modulating the mechanobiology of CAFs.

To understand the mechanism of mechanically driven vascular growth, we chose to inhibit SN1, ROCK, and YAP to test different points along the canonical signaling pathways of actin-myosin contractility in CAFs; this also presented a parallel opportunity to induce expression of a contractility regulator, Rho, in NBFs to promote a “CAF-like” phenotype. We opted to use shRNA inhibition instead of small molecule inhibitors because the majority of our experiments are co-cultures where off target effects would confound interpretation of results. Recent work has indicated that SN1 is required for transforming growth factor beta induced expression of alpha-smooth muscle actin, a necessary protein for contractile machinery and that SN1 inhibition reduces activity of Rho^34,35^. Additionally, enhanced ROCK signaling is associated with stabilization of SN1^36^. Other groups have demonstrated that an inhibitor against both isoforms of ROCK abrogates YAP signaling and limits cell contraction^37–39^. Our key results indicate that CAFs induce vascularization in a 3D *in vitro* microtissue model via active mechanical deformations induced in the ECM, and that this process is not solely dependent on VEGF signaling. Inhibition of the mechanotransductive pathway, specifically ROCK2 and SN1, decreases vascularization potential of CAFs compared to control lines while still showing more vessel growth than NBF samples. This might indicate compensatory mechanisms downstream of these factors that still promote blood vessel growth. In CAFs with depleted YAP, limited vessel growth is observed (similar to NBF groups), demonstrating a role as a mechanoregulator of CAF-associated vascular growth.

Interestingly, knockdown of ROCK1 shows no inhibition of blood vessel growth despite significantly inhibited ability to deform the ECM. Inhibiting ROCK1 has been shown to directly affect turnover of actin specifically at the cell periphery, while leaving the polymerized actin cytoskeletal structure associated with the nucleus intact^40,41^. Taken in context with our data, this suggests that cells need stable actin stress fibers, but not necessarily enhanced actin polymerization associated with focal adhesions at the cell membrane, to induce deformations in the ECM and ultimately drive blood vessel growth. Furthermore, the results demonstrate that ROCK2 may be the more potent regulator of biomechanically controlled blood vessel growth as regulated by CAFs compared to ROCK1. Western blot analysis of CAF-shROCK2 cells showed only ~50% decrease in protein expression compared to CAF-EV (Fig. S2) despite the fact that the inhibited cells demonstrated significantly different vascularization potential than control cells.

Based on these studies, we do not wish to suggest the importance or priority of any one of these specific proteins (ROCK, SN1, YAP) over the others as key regulators of CAF biomechanics. Rather, we view our work as a compellation, showing that inhibiting mechanotransduction at 4 different locations along a pathway affects the ability of CAFs to deform the ECM and thus abrogates vascularization potential of these cells.

It has become increasingly apparent that cell and ECM mechanics impacts tumor progression, and should be considered in the development of novel treatment strategies. Mechanical force can be described as either shear (parallel to a surface) or normal (perpendicular). While shear forces exist in a viscoelastic material such as tissue, they are due predominantly from fluid flowing over surfaces or through a material (i.e., interstitial flow). Normal forces can be either compressive (reducing volume) or tensile (expanding volume). Compression can occur when the activated tumor stroma produces excessive ECM proteins (causing an increase in local density), excessive cell proliferation (causing a local increase in cell mass^42^), or interstitial fluid flow. Tensile forces, on the other hand, can be generated when neighboring cells contract. As differences in cell concentrations were not a factor in regulating blood vessel in our model and interstitial flow was absent, our *in vitro* system represents an environment to isolate and investigate predominantly tensile forces generated by CAFs and their subsequent role in the formation of blood vessels. Although our system does not include interstitial flow, there is strong evidence supporting interstitial flow-derived forces and their role in vessel growth, as well^5,43,44^.

Our hypothesis that CAF-induced biomechanical forces drive vasculogenesis in the tumor microenvironment relies on the assumption that the ECs present in our tissue models are capable of responding to such stimuli. However, as the objective of this study was to characterize the mechanical behavior of CAFs relative to vascular growth, we have not yet investigated the mechanisms that drive the EC response to mechanical stimuli. Several reports suggest that ECs may respond to strains with shifts in phenotype, including promotion of migratory behaviors^45,46^. Additionally, other key receptors involved in vascular growth, including VEGFR2 and Notch1, have been implicated as potential mechanosensors in ECs^47–49^. Furthermore, the response of ECs to shear forces has been extensively studied and may also play a role in vasculature formation^5,7,50–52^. Finally, there may be some influence on EC proliferation promoted by growth factors secreted by CAFs. While we cannot rule out this effect entirely in our studies, we have demonstrated that the mechanical behavior of CAFs does contribute to vascular growth and shown that mechanical perturbations in the absence of stromal cells can promote vascularization.

The remodeling of the peritumoral microenvironment occurs through multiple time-scales, ranging from rapid (on the order of seconds to minutes) fluctuations of the cellular edges via focal adhesion protrusions^53^, to systemic remodeling and deposition of new ECM proteins that occurs over the course of weeks or longer^54–56^. While integrin binding to the ECM occurs on the order of a few minutes, the downstream signaling required to form a mature focal adhesion associated with the contractile actin cytoskeletal structure occurs on the order of an hour^57^, prompting our choice for the timescale of our bead displacement measurements. Thus, our study focused on an intermediate time point where CAFs have induced displacements in the microenvironment, generated through actin-myosin contractile machinery, but have not yet produced significant new ECM deposition or remodeling. As a result, we cannot rule out a role for shorter term (rapid focal adhesion turnover) and longer term (ECM remodeling) events that may apply additional forces to the surface of ECs to impact vascular growth.

The study presented here illustrates how deformations in the ECM induced by CAFs regulate the growth of vasculature in a manner not solely dependent on soluble signaling factors, including VEGF. Further, the studies utilizing magnetic beads demonstrate that ECs respond to direct mechanical stimulation by initiating a vasculogenic process, in the absence of secreted factors produced by CAFs. Based on our magnetic bead studies, we demonstrate that the movements induced in the ECM by mechanical perturbations partially recapitulate vascular growth driven by CAF biomechanics. Furthermore, our magnetic bead studies recapitulate ECM deformations on a similar length scale to those induced by CAFs (4–6 μm), or roughly 25% of the diameter of the average cell. For our static bead displacement experiments, these deformation scales do not represent the dynamic or time-varying features of strains in the ECM, rather they capture a mean tensile strain induced over the observed time period. In contrast, our moving magnetic bead experiments attempted to capture the dynamic behavior of mechanotranductive signaling.

Our studies demonstrate the role of active mechanical forces, generated by CAFs as a regulator of blood vessel growth in a 3D *in vitro* assay of vasculogenesis. While vasculogenic vessel growth was previously believed to occur only during embryogenesis^58–60^, we now understand that multiple cell types, including endothelial progenitor cells, bone marrow stromal cells, tumor cells, and CAFs can promote vasculogenesis in association with the progression of cancer. Our work supports the premise that CAFs can enhance vascular growth through a vasculogenic process that is mediated, in part, by biomechanical forces and that the Rho/ROCK pathway and YAP signaling are involved. Although all of our studies were conducted in an *in vitro* assay, we believe this work correlates with previous *in vivo* work describing the mechanical activity of CAFs as it relates to SN1 signaling in tumor progression^61^. Ultimately, we believe this information will help elucidate potential novel targets for anti-cancer therapeutic strategies designed to modulate the biomechanical behaviors of CAFs that contribute to tumor progression.

## Methods

### Vasculogenic ring assay

To form vascular networks in 3D gels, a 1:1 ratio of endothelial cell colony forming cell-derived endothelial cells (ECFC-ECs or just ECs) derived from umbilical cord blood and fibroblasts were embedded in fibrin gels (10 mg/mL, except where noted). NBFs and CAFs were previously isolated and immortalized utilizing insertion of hTERT into patient-derived fibroblasts^27,62,63^. NHLFs were obtained from Lonza (#CC-2512); multiple donors were utilized throughout the studies between passages 4–8. Fibroblasts were cultured in DMEM (ThermoFisher, #11995065) with 10% FBS (ThermoFisher #26140079), 1% L-glutamine (ThermoFisher, #25030149), 1% non-essential amino acids (ThermoFisher, #11140-050), 1% sodium pyruvate (ThermoFisher #11360070), and 1% penicillin/streptomycin (ThermoFisher, #15140-122). Characterization of NBFs and CAFs has been completed previously in our lab^61,64^. ECs were isolated and derived as described previously from umbilical cord blood samples^65^, cultured in EGM-2 (Lonza, #CC-3124), and utilized between passages 3–6. Microtissues were created by dissolving fibrinogen (Sigma, #8630) in DPBS, and then mixing with cell suspensions to create final concentrations of 5 × 10^4^ of each cell type per gel. Thrombin was added to a final concentration of 3 U/mL before the suspension was plated into a 1 cm diameter poly(dimethyl siloxane) ring on a glass coverslip. Gels were allowed to solidify for a minimum of 20 min before the addition of EGM-2. To test gel composition, fibrinogen concentration was varied and in some studies collagen I (BD Biosciences, #354249) was added to the solution prior to the addition of thrombin. Media was changed every 2 days and experiments were halted via fixation at the end of 7 days.

Vessel growth was analyzed by staining the hydrogels for the endothelial marker CD31 (Dako #M0823, 1:200). Briefly, gels were rinsed with PBS, fixed at −20 °C for 20 min with a 1:1 acetone-methanol mixture, rinsed, blocked for 1 h at room temperature with 2% bovine serum albumin (BSA) in PBS containing 0.1% Tween-20. Antibodies were diluted in the same solution and incubated overnight at 4 °C (Secondary: AlexaFluor-488 Rabbit-anti-Mouse, Invitrogen #A11059, 1:500). Images were acquired on an Olympus IX83 inverted microscope and analyzed using AngioTool software to quantify vessel length^66^. Soluble VEGF produced during the ring assay was measured using VEGF Human ELISA Kit (Thermo Fisher, #KHG0111); a second series of VEGF measurements were taken from samples containing only fibroblasts at the same concentration used in the ring assay. In some experiments, VEGF signaling was inhibited by adding a small molecule inhibitor of VEGFR, SU-5402, (Abcam, ab141368, 1 μM) to the media after 3 days of culture. To analyze conditioned media, 100% conditioned media from ring assays containing both CAFs/ECs or NHLFs/ECs was harvested, spun down at 1000 rpm for 5 min to remove cell debris, and then fed directly to ring assays containing NBFs and ECs.

### ECM displacement analysis

To analyze the mechanical behavior of fibroblasts, we developed a bead displacement algorithm in MATLAB™. Bead displacement events were interpreted as ECM deformations induced by cell contractility events. Using the vasculogenic ring assay setup, 1 μm diameter blue fluorescent polystyrene microbeads (Invitrogen, #F8815) were included as fiducial markers in gels containing 5 × 10^4^ fibroblasts only. Bead movement was tracked in 3D over the course of 1 h, after gels had been incubated overnight. This algorithm thus captured long-term ECM deformations introduced by cell behavior.

### Disruption of contractility pathways

To interrogate the mechanotransductive pathways involved in mechanically regulated vessel growth, we utilized shRNA to inhibit CAF contractility. Specifically, we focused on the Rho/ROCK pathway, which are potent regulators of the actin cytoskeletal structure^67,68^ and YAP, which has been shown to be involved with CAF mechanosensitive behavior^23,37,38^. We also chose to inhibit SN1, a transcription factor shown to be upregulated in CAFs compared to normal fibroblasts^69^, that has recently been shown to be involved in CAF mechanotransduction^34,61^. In a parallel study, we were able to promote enhanced contractility of NBFs using a constitutively active form of Rho inserted into the cells via lentiviral transduction (referred to as NBF-caRho cells). shRNAis were from the Broad collection (Washington University Genome Center). There were 5–7 shRNAis initially selected for each target. These were all screened and 2 shRNAis were chosen. All shRNAis and constitutively active RhoA(Q63L)-Flag tagged cDNA were subcloned into the pLVX-hygro vector. Lentiviruses were produced in HEK 293T cells, and human CAFs were transduced and selected in 200 μg/mL hygromycin. To check knockdown efficiency, cells were lysed in 1X RIPA buffer supplemented with protease inhibitors. Protein lysates were processed following standard procedures. shRNAi target sequences as well as antibody description and working dilutions used can be found in Supplemental Tables S2 and S3; targets were verified previously in our lab^16,36,61^; for these studies, the targeting sequence listed as letter A in Table S3 was utilized.

### Magnetic mechanical stimulation

To eliminate signaling of soluble factors that promote vessel growth, a magnetic bead assay was developed as a means to mechanically stimulate endothelial cells. Iron oxide microbeads (Dynabeads,Thermo Fisher, #14013) with a tosyl-coated surface were mixed with 50 U/mL thrombin in 0.1% BSA in PBS to create thrombin-coated magnetic beads^70^. Beads were added to the vasculogenic ring assay. To stimulate movement, rare earth magnets (N52, B_r_ = 1.32 T) were placed directly adjacent to the well plate containing rings for static magnetic studies. For dynamic stimulation studies, the magnets were located on an orbital shaker 3 cm from the bottom of the well plate. Speed of rotation was varied to adjust magnetic stimulation.

### Statistical analysis

Vasculogenic assays represent a minimum of 3 biological replicates. Bead displacement results represent average magnitudes of displacement based on a minimum of 40 beads analyzed per gel over a minimum of 2 technical replicates. Average displacement vector magnitudes were compared among sample groups. All data was compared via ANOVA, followed by post-hoc Holm-Sidak tests as appropriate with α = 0.05. Unless noted, all data is presented as average ± standard error of the mean, except for deformation data which is presented as average deformation magnitude ± standard deviation.

### Data availability

The datasets generated during and/or analyzed during the current study are available from the corresponding author on request.

## Electronic supplementary material

Supplemental Information
