# Supplementary material for: Cancer-associated fibroblasts support vascular growth through mechanical force
Source: Sci Rep. 2017 Oct 3;7:12574. doi: 10.1038/s41598-017-13006-x (PMC5626692; doi:10.1038/s41598-017-13006-x)
Supplement: Supplementary file 1 — Supplemental Information [file 41598_2017_13006_MOESM1_ESM.pdf]

Supplemental Information:

Cancer-associated fibroblasts support vascular growth through mechanical force

Mary Kathryn Sewell-Loftin[1,5], Samantha Van Hove Bayer[2,5], Elizabeth Crist[1], Taylor Hughes[1], Sofia M. Joison[3], Gregory D. Longmore[2,4,5], and Steven C. George[6 *]

Departments of Biomedical Engineering[1], Cell Biology and Physiology[2], Computer Science and Engineering[3], and Medicine, Oncology Division[4], Washington University in St. Louis, St. Louis, MO 63130; ICCE Institute at Washington University[5]; Department of Biomedical Engineering[6], University of California, Davis, Davis, CA 95616.

*Corresponding Author:

Steven C. George, M.D., Ph.D.

Professor

Department of Biomedical Engineering

University of California, Davis

451 E. Health Sciences Drive

GBSF, Room 2303

Davis, California 95616

Telephone: (949) 910-9195

Email: scgeorge@ucdavis.edu

Supplemental Figures

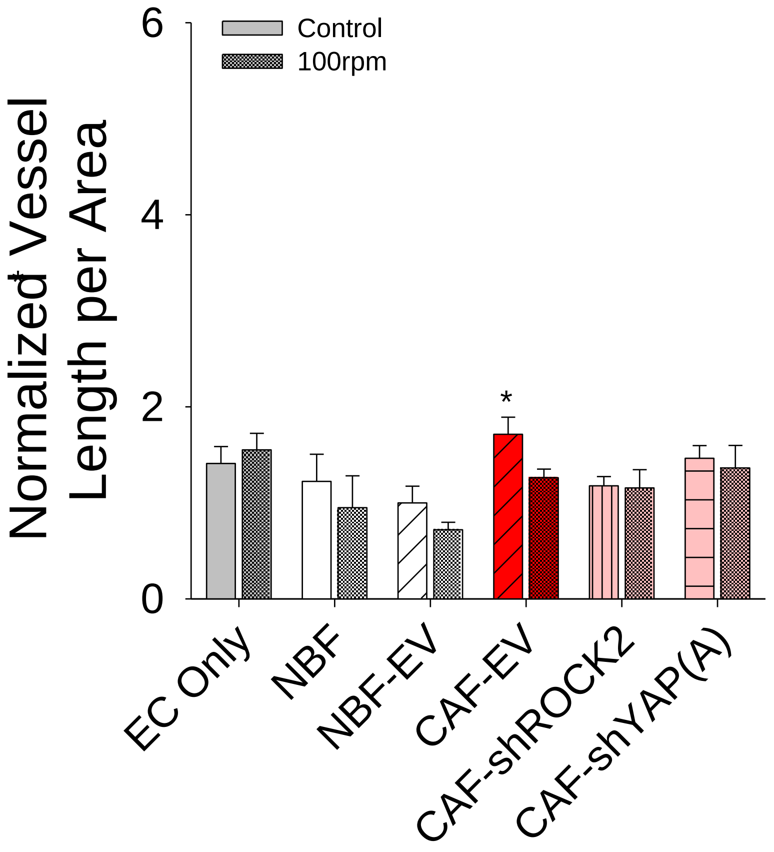

Fig. S1. Limitation of magnetically stimulated vessel growth. Increasing the speed of dynamic stimulation of thrombin-coated magnetic beads to 100 rpm does not promote enhanced blood vessel growth in any cell line tested. Vascular growth numbers are normalized to NBF-EV control: 0.0033 ± 0.0006 μm^{-1}.

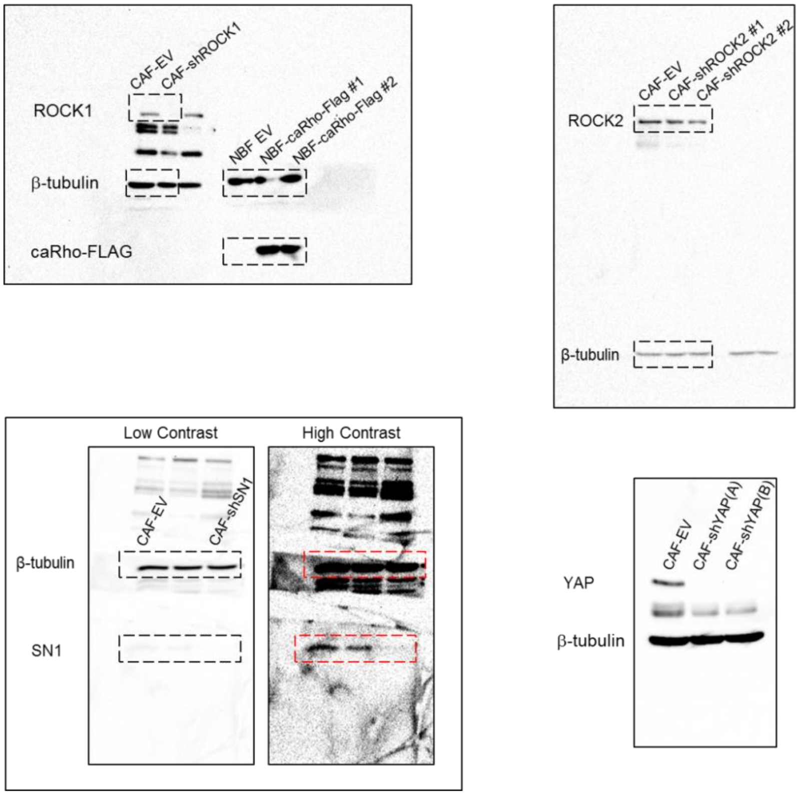

Fig. S2. Protein expression in shRNA treated cell lines. To verify shRNA knockdown of the mechanotransductive pathways in CAFs, Western Blots were completed comparing modified lines to empty vector (EV) controls. Decreases in ROCK1, ROCK2, SN1, and YAP were observed. To verify insertion of Flag-tagged caRho into NBFs, Western Blot analysis was completed. There is a definitive increase in the expression of Flag-tagged caRho observed. Targeting sequences and antibody information can be found in Table S2 and Table S3, respectively. Dashed black lines represent areas of interest on each gel. For SN1 analysis, low and high contrast images are shown; red dashed lines on High Contrast image are same as black dashed lines on Low Contrast image. Note: The rightmost column on the ROCK1 blot as well as the middle column of the SN1 blot was not utilized in any of the studies presented in this manuscript, as it was an unrelated sample.

Table S1. Average deformations by NBFs, CAFs, and NHLFs in different composition of fibrin gels.

**Cell Line**	**Fibrin Concentration (mg/mL)**	**Average Deformation (μm)**
	2.5	7.4 ± 4.6
NBF	5.0	4.9 ± 1.3[@]
	10	2.5 ± 0.8[+]
	2.5	17.3 ± 6.8*
CAF	5.0	6.7 ± 2.5*^
	10	3.4 ± 1.8*^[&]
	2.5	6.3 ± 3.3*[#]
NHLF	5.0	5.4 ± 3.6[#$]
	10	3.9 ± 3.7*[$%]

Table S1. Average deformations induced by NBFs, CAFs, and NHLFs in fibrin gels of different compositions. Values represent averaged magnitudes of deformation vectors ± standard deviation (n > 40). ANOVA was used to compare between groups, followed by post-hoc Holm-Sidak tests as appropriate. There is no difference between NBF and NHLF at 5.0 mg/mL (p = 0.1) or CAF and NHLF at 10 mg/mL (p = 0.1). Other statistical comparisons are as follows: * p < 0.01 vs. NBF at same fibrin concentration;
# p < 0.01 vs. CAF at same fibrin concentration.

For the Significant Decrease in NBF-Generated Deformations in Increasing Fibrin Concentrations –
@ p< 0.01 vs. NBF at 2.5 mg/mL; + p < 0.01 vs. NBF at 5.0 mg/mL

For the Significant Decrease in CAF-Generated Deformations in Increasing Fibrin Concentrations – ^ < 0.01 vs. CAF at 2.5 mg/mL; & < 0.01 vs. CAF at 5.0 mg/mL

For the Significant Decrease in NHLF-Generated Deformations in Increasing Fibrin Concentrations – $ p < 0.01 vs. NHLF at 2.5 mg/mL fibrin; % p < 0.01 vs. NHLF at 5 mg/mL fibrin

Table S2. Antibodies used in shRNA validation and immunofluorescent studies.

Antibody	Company	Catalog number	Dilutions
Alexa Fluor 555 Goat anti-Mouse	Invitrogen	A-11001	IF 1:500
anti-Flag tag antibody	Sigma	F3165, clone M2	WB 1:5000
beta-Tubulin	Sigma	T4026, clone TUB 2.1	WB 1:5000
CD31	Dako	MO82301-2, clone JC70A	IF 1:200
ROCK1	BD Biosciences	#611136	WB 1:1000
ROCK2	BD Biosciences	#610623	WB 1:1000
SNAIL1	Cell Signaling Technology	#3879, clone C15D3	WB 1:1000
YAP	Cell Signaling Technology	#4912	WB 1:1000

Table S3. Targets for shRNA knockdown in mechanotransductive pathways.

Endogenous gene specific	Target Sequence 5' -3'
human ROCK1	A – GAGGTAAATGAACACAAAGTA
	B – GTGGAGATCTTGTAAACTTAA
human ROCK2	A – GCACAGTTTGAGAAGCAGCTA
	B – GCCTTGCATATTGGTCTGGAT
human SNAIL1	A – CCAGGCTCGAAAGGCCTTCAA
	B – CCAAGGATCTCCAGGCTCGAA
human YAP	A – CCATGAACCAGAGAATCA
	B – TAGCTCAGATCCTTTCCT
